# Developing a high-performance liquid chromatography fast and accurate method for quantification of silibinin

**DOI:** 10.1186/s13104-019-4774-2

**Published:** 2019-11-14

**Authors:** Faezeh Bakhshi, Ommoleila Molavi, Mohammad Reza Rashidi, Ali Shayanfar, Hassan Amini

**Affiliations:** 1 Faculty of Pharmacy, Tabriz University of Medical Sciences, Tabriz, Iran; 2 Drug Applied Research Center, Tabriz University of Medical Sciences, Tabriz, Iran; 3 Pharmaceutical Biotechnology Department, Faculty of Pharmacy, Tabriz University of Medical Sciences, Tabriz, Iran; 4 Pharmaceutical Analysis Research Center and Faculty of Pharmacy, Tabriz University of Medical Sciences, Tabriz, Iran; 5 Student Research Committee, Tabriz University of Medical Sciences, Tabriz, Iran; 6 Stem Cell Research Center, Tabriz University of Medical Sciences, Tabriz, Iran; 7 Research Center for Pharmaceutical Nanotechnology, Tabriz University of Medical Sciences, Tabriz, Iran

**Keywords:** Analysis method, HPLC, Silibinin, Method validation, Precision and accuracy

## Abstract

**Objective:**

Silibinin is an antioxidant agent and is shown to have anticancer effects in different cancers including lung, breast, colorectal, liver, prostate, and kidney. There are challenges in the clinical use of silibinin. The main limitation is low solubility, poor oral absorption, and extensive hepatic metabolism. We aim to develop a High-Performance Liquid Chromatography (HPLC) sensitive method for quantification of silibinin in aqueous samples to quantify its concentration in new formulations. A reverse-phase high-performance liquid chromatography (RP-HPLC) composed of C18 column as stationary phase and the mixture of methanol (90%) and water (10%) as mobile phase. The developed method was validated based on the established guidelines.

**Results:**

The retention time for silibinin was seen in 2.97 min after injection. The calibration curve was drawn and the established method demonstrated a linear ranged from 10 to 100 µg/ml, with a correlation coefficient of 0.996. The sensitivity of the developed method was 10 µg/ml. The accuracy calculated in the range of 88–105.9% and the precision (as relative standard deviation) was between 2.7 and 10.9%. These results demonstrate that the developed method can be a fast and accurate method for quantification of silibinin in aqueous samples.

## Introduction

The efficacy of every anticancer agent is usually assessed by its ability to kill tumor cells without damaging normal tissues. The main barrier to utilizing almost all common chemotherapy agents is their toxicity. Two important approaches to develop a safe therapeutic strategy for cancers are (1) Using an anticancer drug with exclusive anticancer effects and (2) Making anticancer drugs target-oriented about tumour which results in a selective concentration of these drugs in tumour sites.

Silibinin is a natural, non-toxic compound with anticancer properties in different cancer models, in vitro and in vivo and is known for its pharmaceutical application and strong antioxidant properties [1–3] and as a polyphenol compound, it has low solubility (Additional file 1: Fig S1). It is prescribed in the form of a capsule and can be absorbed through the digestive system. It conjugates in the liver and is preliminarily defecated by the biliary system [4].

Silibinin has anticancer activities against lung, breast, colon, skin, liver, bladder, prostate, and kidney cancers. Although it is believed that anticancer properties of silibinin are related to its effects on cell proliferation, apoptosis, inflammation, angiogenesis, and metabolism, its accurate biochemical anticancer mechanism is still under investigation [4–13]. In some investigations, silibinin decreases micro-vessel density in prostate carcinoma tumours by decreasing vascular endothelial growth factor (VEGF) expression. Others found that silibinin targeted the onset of angiogenesis in prostate cancer and inhibited signalling originated by Hypoxia-inducible factor 1-alpha (HIF-1α) [14, 15]. In terms of colorectal cancers, silibinin also inhibited angiogenesis through a decrease in expression and production of VEGF, HIF-1α، cyclooxygenase-II (COX2), and inducible nitric oxide synthase (iNOS) [16]. Recent investigations have also identified silibinin to target genes and pathways in different cancers (Additional file 2: Table S1) [13–18].

A fundamental reason to choose silibinin in developing targeted anticancer nanoparticles is its low toxicity in humans. On the other hand, the main limitation in the clinical application of silibinin to treat cancer is its poor solubility that lowers its bioavailability which affects its clinical application [19, 20]. The main barrier to the clinical application of silibinin in treating cancer is its low solubility and bioavailability. A key and successful strategy to enhance anticancer drugs solubility and their tumour targeting properties are to use polymer nanoparticles (nanocarriers) capable of carrying a wide range of anticancer drugs [3, 21–26].

We aim to develop an HPLC–UV method, common technique for analysis of pharmaceuticals, for quantification of silibinin in the aqueous samples in order to quantification of its concentration in in vitro experiments of new formulations.

## Main text

### Materials and methods

#### Chemicals

Silibinin with a purity degree of ˃ 98% (Sigma Aldrich, Catalogue No: S0417), acetonitrile, and methanol that were used in this study were from Merck Corporation (Germany). Distilled deionized water was from Ghazi Corporation (Tabriz, Iran).

#### Chromatographic condition

Waters HLPC system (Milford MA, USA) is comprised of one Waters 515 pump, Waters 2487 Ultra Violet (UV) detector, and Empower Pro 2002 Waters V.500 software to analyze of silibinin. A mixture of 90% methanol and 10% distilled water was used as mobile phase. This solution was degassed in a sonicator (Transonic T420Elma, Germany) at the pre-examination phase. The used column for analysis was Eurosphor C18 column, with protective column (5 mm, 250 × 4.6 mm, KNAUER, Germany). The flow rate was adjusted at 1 ml/min, injecting 20 µl of samples by Waters 717Plus auto-sampler. Silibinin was detected by UV detector at 288 nm wavelength. The room temperature was 25 °C.

The stock solution (1 mg/ml) was prepared through solving an adequate amount of silibinin powder in methanol. The solution was kept in the refrigerator for examinations. The daily solution was diluted up to the concentration ranged from 10 to 100 µg/ml, with each concentration to be kept in 2 ml micro-tube, which then was injected into the system.

#### Analysis method

C18 column was used as the stationary phase. A mixture of water with methanol and acetonitrile to select mobile phase. In every mentioned mobile phase, silibinin was injected to the system, and then according to the shape of the peak and repeatability, the appropriate results of the mobile phase were selected.

#### Analytical method validation

Validation of a developed method includes linear equation assessment, and findings accuracy and precision. Linear equations were studied using calibration norms resulted from silibinin stock solution data in aqueous samples with 10, 20, 40, 60, 80, 100 µg/ml concentrations.

The lowest and highest concentrations of the standard curve were respectively demonstrated as the lower limit of quantification (LLOQ) and upper level of quantification (ULOQ). American Food and Drug Administration (FDA) suggested LLOQ for the assessment of developed parameters sensitivity. Nevertheless, other parameters for the assessment of the sensitivity of developed quantification methods are limit of detection (LOD) and limit of quantification (LOQ).

Inter-day and intra-day accuracy and precision of developed quantification method were studied by the preparation of 3 samples for quality assessment including three aqueous samples at 15, 50, and 75 µg/ml concentrations that were in calibration range.

#### Presenting an analysis method and drawing a calibration curve

A mixture of methanol and water with 90:10 ratio was used as mobile phase to identify silibinin (as a non-ionized drug) in aqueous solution, and the flow rate of mobile phase was adjusted to 1 ml/min. Obtained UV results reveals that the UV signals were enhanced correspondingly by increasing the concentration of silibinin. According to UV results to different injected concentrations (from 10 to 100 µg/ml) of silibinin, the calibration curve was drawn.

#### Statistical analysis

Average, standard deviation and relative standard deviation were used to evaluation of precision and regression analysis was applied to find correlation between concentration and peak area. The data were analysed using SPSS24 software.

### Results

**Fig. 1:**
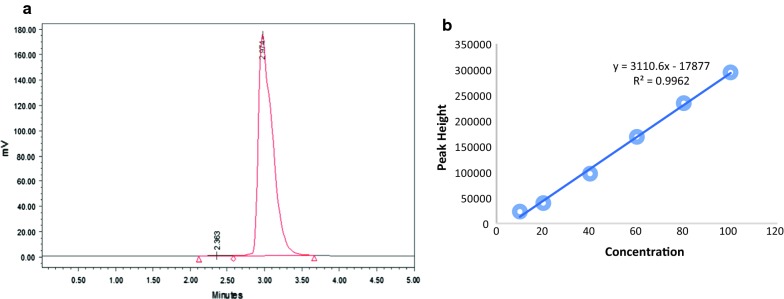
**a** Silibinin chromatogram in aqueous samples with a concentration of 50 µg/ml. **b** Silibinin calibration curve in aqueous samples: the horizontal axis shows the injected silibinin concentrations (µg/ml), and the vertical axis shows peak height of chromatograms)

HPLC–UV diagram for silibinin in aqueous samples showed he retention time in 2.97 min after injection. As the diagram illustrates, symmetric and acute peaks can be observed in injections (Fig. 1a). There was a linear correlation with a coefficient of determination (R^2^) equal with 0.996 between concentration (10 to 100 µg/ml) and height peak. The linear equation and R-squared are depicted in Fig. 1b.

The sensitivity of the developed method (LLOQ) according to FDA guidelines, was 10 µg/ml. Based on ICH guidelines, LOD and LOQ were valid parameters for the sensitivity of aqueous samples and in the developed method were respectively calculated to be 7.25 µg/ml and 24.2 µg/ml (Table 1).

**Table 1:** Linear parameters for in vitro samples

Parameters	Value
Slope	3110.6
Intercept	− 17,877
Standard deviation of calibration curve	7515.2
Linear range (µg/ml)	10–100
Coefficient of determination (R^2^)	0.996
LLOQ (µg/ml)	10
ULOQ (µg/ml)	100
LOD (µg/ml)	7.25
LOQ (µg/ml)	24.2

Calibration curve parameters, linear range, LLOQ, ULOQ, LOD, LOQ for Silibinin in in vitro samples

The results of HPLC–UV of three concentrations (15 µg/ml, 50 µg/ml and 70 µg/ml) in inter-day and intra-day (Additional file 3: Table S2) calculated for evaluation of accuracy and precision of the developed method. The highest accuracy seen in concentration of 50 µg/ml in inter-day evaluations (105.9%) and the lowest accuracy related to concentration of 15 µg/ml in intra-day evaluations (88.0%). The highest and lowest RSD seen in the concentration of 15 µg/ml in intra-day (10.9%) and in the concentration of 75 µg/ml in inter-day (2.7%) calculations, respectively (Table 2).

**Table 2:** Accuracy and precision

Added concentration (µg/ml)	Calculated concentration (µg/ml)	RSD (%)	Accuracy (%)
Inter-day			
15.0	14.3	10.6	95.1
50.0	53.0	3.1	105.9
75.0	75.8	2.7	101.1
Intra-day			
15.0	13.2	10.9	88.0
50.0	50.6	4.0	101.2
75.0	75.4	0.8	100.5

Inter-day and intra-day accuracy and precision of the developed method for in vitro method

### Discussion

Silibinin is a non-toxic herbal product that possesses anticancer properties in different types of cancers, both in vivo and in vitro. Silibinin compounds, besides inducing apoptosis, can target different signalling pathways factors in cells, including transcription factors, growth factors, cell survival factors, inflammatory cytokines, kinase proteins, and angiogenesis factors. These might result in an effective and selective killing of cancer cells. Studies have shown that silibinin has different target effects in cells [27].

Silibinin absorption and metabolism has been studied in different investigations. In the bloodstream, silibinin is more observed in a conjugated form. In several studies, after oral prescription to healthy volunteers, only 10 to 17% of silibinin was observed as a non-conjugated form [28, 29]. Silibinin plasma concentration peak was obtained in mice after 30 min of injection and in tissues in min 60 post-injection. Then, the plasma concentration was reduced with the half-life of 57–127 min. It is while the concentration of conjugated silibinin tends to be reduced with the half-life of 45–94 min [28]. An important issue regarding silibinin metabolism is its fast purgation in both forms of free and conjugated. In a clinical trial, the half-life of silibinin was demonstrated in both forms to be almost 6.3 h. Because of fast metabolism; its plasma concentration is usually in nanomolar (nM) range, while in a few cases, it is in micromolar (µM) range [29].

One of the key reasons for choosing silibinin in developing anticancer targeted nanoparticles is its low toxicity in humans. For instance, in two clinical trials, silibinin phytosome, which is a commercial formulation of silibinin, was orally prescribed to prostate cancer patients in a dose of 13 g/day and for the average of 20 days or 20–25 g/day for the average of 28 days. The results showed that blood silibinin after 1-h post-injection was approximately 20 µM without any severe toxicity for these patients. The lethal dose for intravenous injection in 50% of cases was reported to be 400 mg/kg for mice and 385 mg/kg for rats while other studies reported a safe injection of the drug in relatively higher doses [29].

Nevertheless, there are some challenges in applying silibinin as an anticancer drug. Its separation and purification from its herbal origin, its pharmaceutical issues, long time and high costs of epidemiological studies on this drug, anticancer properties of flavonoids due to their poor solubility, poor oral absorption, and extensive hepatic metabolism are some of these barriers [30]. There are some approaches to overcome these challenges. For instance, enhancers like Piperine, amid alkaloid extracted from *Piperaceae* herbal family can be a solution [31]. To improve the pharmaceutical properties of silibinin, another approach is to use sustained nanoemulsion synthesis technology. In this method, lipophilic flavonoids can be made in the form of emulsions comprised of nanoparticles with very small sizes (< 200 nm). In this manner, flavonoids are gradually released and will have more absorption and biocompatibility after oral prescription [32].

An important issue in pharmaceutical research is to develop a precise, accurate, and repeatable method to quantify a drug in its specific solution in order to control the quality and assess its environmental sustainability, release from specific formulation, and pharmaceutical researches [33]. To quantify a drug in solutions, a common way is chromatography. HPLC is a proper method to separate, measure, and demonstrate a type of material. It is one of the most common methods to quantify drugs in pharmaceutical researches. A great merit of this method is the opportunity to determine the structure and the level of impurity in drugs and formulations. This opportunity is not limited to synthetic drugs and can be applied to herbal drugs, too [34–37].

This study was carried out to establish an experimental process using HPLC, based on previously-determined parameters in HPLC, such as choosing stationary, mobile phase and a detector, demonstrating the rate of mobile phase was utilized. Then, according to FDA guidelines, validation of a developed method, such as evaluation of a linear equation, validity and accuracy of findings, took place. Analysis of calibration curve in concentrations of 10, 20, 40, 60, 80, and 100 µg/ml suggested a linear correlation between concentration and peaks’ height. According to Fig. 1, R^2^ in aqueous samples was 0.996, which reveals the linear connection between different concentrations. Retention time in aqueous solution was 2.97 min that suggested a fast method for the identification of drug in aqueous solutions. Analysis of inter-day and intra-day data (RSD < 10.9%, precision 88–105.9%) showed that the accuracy and precision of the developed method in the quantification of silibinin in aqueous solutions were acceptable.

### Conclusion

The present study developed a simple and fast method to quantify silibinin in the aqueous method. To do this, an HPLC sensitive method to quantify silibinin in aqueous samples was developed. Validation approaches showed high precision and accuracy of the developed method, meaning that it can be applied as a trusted method in the quantification of silibinin in aqueous samples.

## Limitation

While validation of the developed method showed high precision and accuracy of quantification of silibinin in aqueous samples it is needed to establish an extraction or protein precipitation method for its quantification in plasma samples.

## Supplementary information

**Additional file 1: Figure S1.** *Silybum marianum* and its chemical structure.

**Additional file 2: Table S1.** Genes and pathways affected by silibinin.

**Additional file 3: Table S2.** Inter-day and intra-day results.

## Data Availability

The datasets used and/or analysed during the current study are available from the corresponding author on reasonable request.

## Abbreviations

HPLC: high-performance liquid chromatography
RP-HPLC: reverse-phase high-performance liquid chromatography
VEGF: vascular endothelial growth factor
HIF-1α: hypoxia-inducible factor 1-alpha
COX2: cyclooxygenase-II
iNOS: inducible nitric oxide synthase
LLOQ: lower limit of quantification
ULOQ: upper level of quantification
LOD: limit of detection
LOQ: limit of quantification
µM: micromolar

## References

[1] Haddad Y, Vallerand D, Brault A, Haddad PS (2011). Antioxidant and hepatoprotective effects of silibinin in a rat model of nonalcoholic steatohepatitis. Evid Based Complement Altern Med.

[2] Serviddio G, Bellanti F, Stanca E, Lunetti P, Blonda M, Tamborra R (2014). Silybin exerts antioxidant effects and induces mitochondrial biogenesis in liver of rat with secondary biliary cirrhosis. Free Radic Biol Med.

[3] Rezabakhsh A, Fathi F, Bagheri HS, Malekinejad H, Montaseri A, Rahbarghazi R, Garjani A (2018). Silibinin protects human endothelial cells from high glucose-induced injury by enhancing autophagic response. J Cell Biochem..

[4] Wu J-W, Lin L-C, Hung S-C, Lin C-H, Chi C-W, Tsai T-H (2008). Hepatobiliary excretion of silibinin in normal and liver cirrhotic rats. Drug Metab Dispos.

[5] Tyagi A, Singh RP, Ramasamy K, Raina K, Redente EF, Dwyer-Nield LD (2009). Growth inhibition and regression of lung tumors by silibinin: modulation of angiogenesis by macrophage-associated cytokines and nuclear factor-κB and signal transducers and activators of transcription 3. Cancer Prevent Res.

[6] Dastpeyman M, Motamed N, Azadmanesh K, Mostafavi E, Kia V, Jahanian-Najafabadi A (2012). Inhibition of silibinin on migration and adhesion capacity of human highly metastatic breast cancer cell line, MDA-MB-231, by evaluation of β1-integrin and downstream molecules, Cdc42, Raf-1 and D4GDI. Med Oncol.

[7] Kauntz H, Bousserouel S, Gosse F, Marescaux J, Raul F (2012). Silibinin, a natural flavonoid, modulates the early expression of chemoprevention biomarkers in a preclinical model of colon carcinogenesis. Int J Oncol.

[8] Kaur M, Velmurugan B, Tyagi A, Agarwal C, Singh RP, Agarwal R (2010). Silibinin suppresses growth of human colorectal carcinoma SW480 cells in culture and xenograft through down-regulation of β-catenin-dependent signaling. Neoplasia.

[9] Varghese L, Agarwal C, Tyagi A, Singh RP, Agarwal R (2005). Silibinin efficacy against human hepatocellular carcinoma. Clin Cancer Res.

[10] Lee M-H, Huang Z, Kim DJ, Kim S-H, Kim MO, Lee S-Y (2013). Direct targeting of MEK1/2 and RSK2 by silybin induces cell-cycle arrest and inhibits melanoma cell growth. Cancer Prevent Res.

[11] Singh RP, Tyagi A, Sharma G, Mohan S, Agarwal R (2008). Oral silibinin inhibits in vivo human bladder tumor xenograft growth involving down-regulation of survivin. Clin Cancer Res.

[12] Deep G, Gangar SC, Rajamanickam S, Raina K, Gu M, Agarwal C (2012). Angiopreventive efficacy of pure flavonolignans from milk thistle extract against prostate cancer: targeting VEGF-VEGFR signaling. PLoS ONE.

[13] Liang L, Li L, Zeng J, Gao Y, Chen Y-L, Wang Z-Q (2012). Inhibitory effect of silibinin on EGFR signal-induced renal cell carcinoma progression via suppression of the EGFR/MMP-9 signaling pathway. Oncol Rep.

[14] Singh RP, Raina K, Sharma G, Agarwal R (2008). Silibinin inhibits established prostate tumor growth, progression, invasion, and metastasis and suppresses tumor angiogenesis and epithelial-mesenchymal transition in transgenic adenocarcinoma of the mouse prostate model mice. Clin Cancer Res.

[15] Deep G, Kumar R, Nambiar DK, Jain AK, Ramteke AM, Serkova NJ (2017). Silibinin inhibits hypoxia-induced HIF-1α-mediated signaling, angiogenesis and lipogenesis in prostate cancer cells: in vitro evidence and in vivo functional imaging and metabolomics. Mol Carcinog.

[16] Singh RP, Gu M, Agarwal R (2008). Silibinin inhibits colorectal cancer growth by inhibiting tumor cell proliferation and angiogenesis. Cancer Res.

[17] Nishida N, Yano H, Nishida T, Kamura T, Kojiro M (2006). Angiogenesis in cancer. Vasc Health Risk Manag.

[18] Tiwari P, Mishra K (2015). Silibinin in cancer therapy: a promising prospect. Cancer Res Front.

[19] Zhao J, Agarwal R (1999). Tissue distribution of silibinin, the major active constituent of silymarin, in mice and its association with enhancement of phase II enzymes: implications in cancer chemoprevention. Carcinogenesis.

[20] Wing Ying Cheung C, Gibbons N, Wayne Johnson D, Lawrence Nicol D (2010). Silibinin-a promising new treatment for cancer. Anticancer Agents Med Chem.

[21] Mahmud A, Xiong X-B, Aliabadi HM, Lavasanifar A (2007). Polymeric micelles for drug targeting. J Drug Target.

[22] Ferrari M (2005). Cancer nanotechnology: opportunities and challenges. Nat Rev Cancer.

[23] Jain K (2005). Nanotechnology-based drug delivery for cancer. Technol Cancer Res Treat.

[24] Malam Y, Loizidou M, Seifalian AM (2009). Liposomes and nanoparticles: nanosized vehicles for drug delivery in cancer. Trends Pharmacol Sci.

[25] Aliabadi HM, Shahin M, Brocks DR, Lavasanifar A (2008). Disposition of drugs in block copolymer micelle delivery systems. Clin Pharmacokinet.

[26] Haddadi R, Brooshghalan SE, Farajniya S, Nayebi AM, Sharifi H (2015). Short-Term Treatment with Silymarin Improved 6-OHDA-Induced Catalepsy and Motor Imbalance in Hemi-Parkisonian Rats. Adv Pharm Bull..

[27] Millimouno FM, Dong J, Yang L, Li J, Li X (2014). Targeting apoptosis pathways in cancer and perspectives with natural compounds from mother nature. Cancer Prevent Res.

[28] Flaig TW, Gustafson DL, Su L-J, Zirrolli JA, Crighton F, Harrison GS (2007). A phase I and pharmacokinetic study of silybin-phytosome in prostate cancer patients. Invest New Drugs.

[29] Loguercio C, Andreone P, Brisc C, Brisc MC, Bugianesi E, Chiaramonte M (2012). Silybin combined with phosphatidylcholine and vitamin E in patients with nonalcoholic fatty liver disease: a randomized controlled trial. Free Radic Biol Med.

[30] Amawi H, Ashby CR, Tiwari AK (2017). Cancer chemoprevention through dietary flavonoids: what’s limiting?. Chin J Cancer.

[31] Tatiraju DV, Bagade VB, Karambelkar PJ, Jadhav VM, Kadam V (2013). Natural bioenhancers: an overview. J Pharmacogn Phytochem.

[32] Macedo AS, Quelhas S, Silva AM, Souto EB (2014). Nanoemulsions for delivery of flavonoids: formulation and in vitro release of rutin as model drug. Pharm Dev Technol.

[33] Siddiqui MR, AlOthman ZA, Rahman N (2017). Analytical techniques in pharmaceutical analysis: a review. Arab J Chem.

[34] Hassan B (2012). HPLC uses and importance in the pharmaceutical analysis and industrial field. Pharmaceut Anal Acta.

[35] Mowafy HA, Alanazi FK, El Maghraby GM (2012). Development and validation of an HPLC–UV method for the quantification of carbamazepine in rabbit plasma. Saudi Pharm J.

[36] Islambulchilar Z, Ghanbarzadeh S, Emami S, Valizadeh H, Zakeri-Milani P (2012). Development and Validation of an HPLC Method for the Analysis of Sirolimus in Drug Products. Adv Pharm Bull..

[37] Danafar H, Hamidi M (2015). Simple and Sensitive High-Performance Liquid Chromatography (HPLC) Method with UV detection for mycophenolic acid assay in human plasma. Application to a Bioequivalence Study. Adv. Pharm Bull..

