# Supplementary figures and images for: Developing a high-performance liquid chromatography fast and accurate method for quantification of silibinin
Source: BMC Res Notes. 2019 Nov 14;12:743. doi: 10.1186/s13104-019-4774-2 (PMC6854794; doi:10.1186/s13104-019-4774-2)

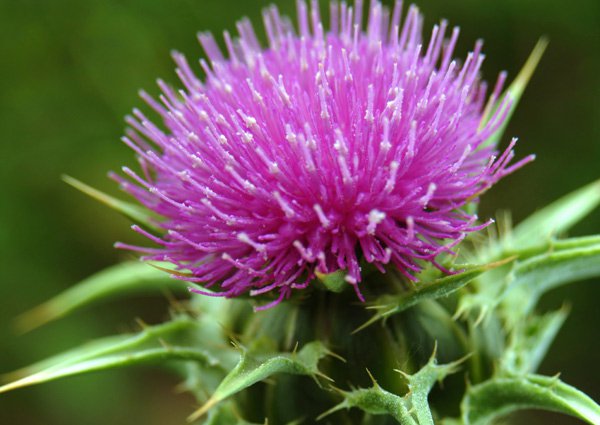

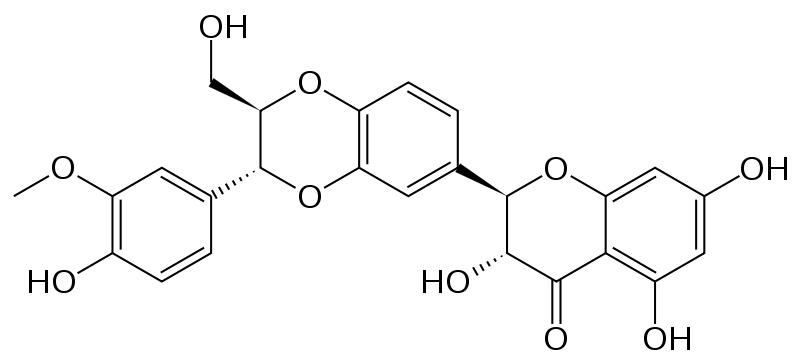


**Fig S1:** Silybum marianum and its chemical structure

Supplement: Supplementary file 1 — Additional file 1: Figure S1. Silybum marianum and its chemical structure. [file 13104_2019_4774_MOESM1_ESM.docx]
