# Supplementary material for: Developing a high-performance liquid chromatography fast and accurate method for quantification of silibinin
Source: BMC Res Notes. 2019 Nov 14;12:743. doi: 10.1186/s13104-019-4774-2 (PMC6854794; doi:10.1186/s13104-019-4774-2)
Supplement: Supplementary file 2 — Additional file 2: Table S1. Genes and pathways affected by silibinin. [file 13104_2019_4774_MOESM2_ESM.docx]

**Table S1.** Genes and pathways affected by silibinin

| **Cancer** | **Identified pathways in inhibiting angiogenesis** |
| --- | --- |
| Colon | Affecting angiogenesis and metastasis through COX-1, COX-2, VEGF, NOS, HIF-1α pathways |
| Prostate | Affecting angiogenesis and metastasis through KRT18, MMP-2, VEGF, VIM pathways |
| Breast | Affecting angiogenesis and metastasis through COX-2, MMP-2, VEGF pathways |
| Lung | Affecting angiogenesis and metastasis through COX-1, COX-2, VEGF, NOS pathways |
| Bladder | Affecting angiogenesis and metastasis through MMP-2, KRT18, KRT19, VIM pathways |

Identified genes and pathways in different cancers affected by silibinin in inhibiting angiogenesis
