# Supplementary material for: Developing a high-performance liquid chromatography fast and accurate method for quantification of silibinin
Source: BMC Res Notes. 2019 Nov 14;12:743. doi: 10.1186/s13104-019-4774-2 (PMC6854794; doi:10.1186/s13104-019-4774-2)
Supplement: Supplementary file 3 — Additional file 3: Table S2. Inter-day and intra-day results. [file 13104_2019_4774_MOESM3_ESM.docx]

**Table S2.** Inter-day and intra-day results

| **Concentration (µg/ml)** | **Peak Height (HPLC)** | | |
| --- | --- | --- | --- |
|  | 1^st^ Day | 2^nd^ Day | 3^rd^ Day |
| 15 | 29414 | 35971 | 37900 |
| 15 | 39930 | 35636 | 42584 |
| 15 | 34712 | 35554 | 47296 |
|  |  |  |  |
| 50 | 133095 | 152028 | 168641 |
| 50 | 158750 | 152693 | 161229 |
| 50 | 164241 | 151207 | 159002 |
|  |  |  |  |
| 75 | 211354 | 225890 | 293427 |
| 75 | 245021 | 233954 | 304578 |
| 75 | 246020 | 234094 | 304570 |
